# The Victory Parade and After

**Published:** 1919-08-02

**Authors:** Henry C. Burdett


### THE EDITOR'S LETTER-BOX.

### THE VICTORY PARADE AND AFTER.

### SOMETHING WORTH ATTENTION BY EACH MAN AND WOMAN.

Peace Day was worthily kept, and July 19 should be held in everlasting remembrance throughout the nation and Empire. On that day we were all united and of one mind. I spent the day in study and observation. The open spaces, the parks of the Metropolis were never so fully peopled or so blessed in fulfilment. Everywhere the people of all classes mingled happily together, and everywhere there was joy and contentment, bright faces, the exhibition of consideration for each other, and the genuine desire to contribute to the general enjoyment, and a great content for all.

There have been many Victory parades on the termination of wars, but Saturday's was demonstrably the greatest and best the world has ever seen. It was not merely a great spectacle, splendidly managed, where all were duly honoured and acclaimed, but it demonstrated that all participants and people were equally heartened, inspirited, and of good cheer. There was indeed, for those who had eyes to see, more than one great parade, and the most uplifting that of the sailors, soldiers, and all ranks who marched to their quarters after saluting King George. Everyone of them, sailors, soldiers, non-combatants, of all nationalities, marched by with happy vitalised faces and vigorous step. They had fought the good fight against the enemy, disease, and death; they had received the heartfelt recognition and thanks of those for whom they had risked their lives, and whose safety and freedom they had established and won. It was evident all felt and knew this. They were homeward bound with merry, contented hearts. It was not possible to watch these brave and triumphant warriors without inspiration, a belief in the future of the League of Nations and the outlook for the future. Few more grateful sights have ever been witnessed in London than the multitude of family gatherings after Victory Parade, in the Green Park, St. James's and Hyde Parks, where the people made themselves at home and had a good time together, whilst they lunched and were merry. A truly delightful peep was here to be gained and shared.

So much for the morning of a Great Day. But what of the permanently disabled? Amongst the vast crowds of spectators there were people from every part of the country and many sections of the Empire. Amongst them the disabled and wounded, who are the children of the nation, as Parliament has declared and undertaken to provide. In an especial manner they are, too, the neighbours, friends or relatives of the people in the localities which sent them to the war. Is everyone of these brave men, are all their children, adequately provided for? Why should not the people in each locality set up a *Victory Committee* with an annual meeting held on each succeeding July 19, from henceforth, to which its duty will be to report on the progress, present position, and circumstances of the warriors in their centre, who have helped to win the war, who are the children and care of them all? July 19 should be a great day everywhere throughout the country in future years for these noble fellows, their wives and families, and the people amongst whom they live. The Ministry of Pensions under present management is doing much to secure that no such child of the State shall be overlooked or uncared for. But the plan I here suggest for a great outpouring of voluntary personal service on the part of all of us who owe so much to our warriors, whether or not encouraged by municipal and county authorities, should be taken up warmly by the best-hearted people. It would be of great value, and should prove a joy in practice to all who take part in such neighbourly and personal service. So I ask men and women everywhere to decide to act at once. The The Victory Parade was a triumph. Let it be followed by "The Victory Committee" throughout the country.

I know its usefulness, in wise and sympathetic hands, from more than twenty years of personal service, and some possible dangers of the moment. Victory is ours, let us prove worthy of it. Let us keep these devoted children of the State in perpetual remembrance.

(Signed) Henry C. Burdett.

[Correspondence is invited.—Ed. The Hospital.]

